# Mouthguard Use Effect on the Biomechanical Response of an Ankylosed Maxillary Central Incisor during a Traumatic Impact: A 3-Dimensional Finite Element Analysis

**DOI:** 10.3390/life10110294

**Published:** 2020-11-20

**Authors:** Alexandre Luiz Souto Borges, Amanda Maria de Oliveira Dal Piva, Laís Regiane da Silva Concílio, Tarcisio José de Arruda Paes-Junior, João Paulo Mendes Tribst

**Affiliations:** 1 Institute of Science and Technology, São Paulo State University (Unesp), São José dos Campos, São Paulo 12220690, Brazil; alexandre.borges@unesp.br (A.L.S.B.); amanda.piva@unesp.br (A.M.d.O.D.P.); tarcisio.paes@unesp.br (T.J.d.A.P.-J.); 2 Department of Prosthodontics, School of Dentistry, University of Taubaté, Taubaté, São Paulo 12020-340, Brazil; regiane1@yahoo.com

**Keywords:** mouthguard, trauma, finite element analysis, athletic injuries

## Abstract

(1) Background: Trauma is a very common experience in contact sports; however, there is an absence of data regarding the effect of athletes wearing mouthguards (MG) associated with ankylosed maxillary central incisor during a traumatic impact. (2) Methods: To evaluate the stress distribution in the bone and teeth in this situation, models of maxillary central incisor were created containing cortical bone, trabecular bone, soft tissue, root dentin, enamel, periodontal ligament, and antagonist teeth were modeled. One model received a MG with 4-mm thickness. Both models were subdivided into finite elements. The frictionless contacts were used and a nonlinear dynamic impact analysis was performed in which a rigid object hit the model at 1 m·s^−1^. For each model, an ankylosed periodontal ligament was simulated totaling 4 different situations. The results were presented in von-Mises stress maps. (3) Results: A higher stress concentration in teeth and bone was observed for the model without a MG and with ankylosed tooth (19.5 and 37.3 MPa, respectively); the most promising mechanical response was calculated for patients with healthy periodontal ligament and MG in position (1.8 and 7.8 MPa, respectively). (4) Conclusions: The MG’s use is beneficial for healthy and ankylosed teeth, since it acts by dampening the generated stresses in bone, dentin, enamel and periodontal ligament. However, patients with ankylosed tooth are more prone to root fracture even when the MG is in position compared to a healthy tooth.

## 1. Introduction

Ankylosis is the replacement resorption process of periodontal ligament (PDL) after dental trauma, which typically led to a direct contact between alveolar bone and root dentin [1]. Usually, the tooth avulsion is reported as one of the main causes of dentoalveolar ankylosis. However, it also occurs after an aggressive trauma without avulsion, surgical endodontic treatments, and intentional tooth replantation [2,3,4]. The ankylosis can present a progressive behavior, with continuous root external resorptions or internal resorptions, leading to the tooth loss [1,2,4]. From the biomechanical point of view, the dentoalveolar ankylosis is related to the prognosis of the tooth modifying the biological aspects and the mechanical response against chewing forces [5]. A previous study reported that the ankylosed tooth and the surrounding alveolar bone show an inferior biomechanical behavior with higher stress magnitude, suggesting a higher risk of failure during the incidence of masticatory loads [5].

During the dental trauma, the majority of injuries involves the anterior teeth and usually affects only the tooth that suffered the impact [6,7]. It is well reported that during a trauma, the biomechanical response from maxillofacial structures involves the enamel, root dentin, periodontal ligament, and surrounding bone at the load dissipation [8]. However, the mechanical response of an ankylosed tooth was not reported yet. The cause of root resorption of a dentoalveolar ankylosis tooth was not completely investigated; therefore, the present study hypothesized that a maxillofacial traumatic impact involving a central incisor affected by dentoalveolar ankylosis could cause a higher stress concentration than a health incisor with PDL. This hypothesis is based on situations with dental implants, for example, where the absence of PDL generates a different stress pattern with higher magnitude than a natural tooth [5].

Basketball, football, hockey, martial arts, and boxing are some examples of contact sports that present a high risk of dental injuries [9]. The maxillofacial trauma in athletes frequently occurs in younger patients during contact sports activities. However, dental trauma in sports differs from other dental trauma, as it is possible to easily prevent by the use of mouthguards [10]. In addition, awareness of the importance of mouthguards did not mean its usage by the athletes, except by hockey players [10]. In this sense, the literature is very concise regarding the preventive effect of mouthguard device to reduce the incidence of maxillofacial injuries during contact activities [4,7,8,9,10,11,12,13,14,15,16].

Three types of mouthguard are available: boil-and-bite, stock, and custom-made. The boil-and-bite mouthguard is made by a thermoplastic material that should be heated in hot water and then formed in the mouth by pressure. The stock type is prefabricated and cheaper than the other two, similar to “plastic trays” that fit over the teeth without contact all the faces. The custom-made type is individual and made on a model of the patient’s mouth [10,11,12,13,14,15,16]. The beneficial effect of the mouthguard use is even more notable when a custom-made mouthguard device is selected, because it precisely fits the patient arch, reducing the stress during an impact [13]. In addition, custom-made mouthguards show superior mechanical performance, protection, retention, comfort, fit, ease of speech, and breathing than stock mouthguards [17]. However, even with a mouthguard in position, some stress will occur in the maxillofacial structures and can induce mechanical complications [11,16].

Regarding the mouthguards’ manufacturing, polymers are, unquestionably, the appropriate class of materials for the production of mouthguards [18]. Nowadays, ethylene vinyl acetate (EVA) copolymer is the most common polymer used for it. Polyolefin, polyvinyl chloride, polyurethane, acrylic resin, silicone rubbers, and even polyetheretherketone are some examples of materials evaluated in the past as possible to be used; however, none of them behaves as well as EVA for this indication [18]. In the future, the computer-aided design and computer-aided technology as subtractive manufacturing process [19] and the 3D-impression as additive manufacture [18] will provide new possibilities of materials usage and design for mouthguard definition and customization [18,19]. Until then, the custom-made process with EVA continues to be the gold-standard.

The use of finite element analysis (FEA) is a reliable method for assessing strain and stress under load incidence in complex structures through numerical models. Since it is difficult to replicate a maxillofacial trauma in situ, FEA seems to be a pertinent method to identify the potential regions of structural failure during an impact [8,20]. In many cases in medicine and dentistry, where research on the trauma mechanics caused by impact, the results obtained from the FEA are the only available data [21]. This method can also assist in interpreting the biological tissues responses that are affected by the traumatic event [8,20]. Different from the in vitro studies, the FEA allows to observe the mechanical response of each separate structure that compound the traumatized region, identifying how the load will be dissipate through the model. The literature reports that the stress cushion efficiency of the mouthguards vary according to the different thicknesses and impact directions [21], which also justify the use of a numerical simulation to standardize the load application between the different models allowing to compare a similar trauma with and without mouthguard use. Despite the fact that the first reports of using FEA to study mouthguards apply a static structural analysis [22], nowadays the computer-aided engineering software constant update and knowledge about the different structure’s mechanical properties and boundary conditions allow the use of dynamic impact [23,24].

The information regarding whether a patient with a dentoalveolar ankylosed tooth can be properly protected from traumas when wearing a mouthguard is not available in the scientific literature nowadays. Based on this, the present study aimed to investigate the influence of dentoalveolar ankylosis on a central incisor and the surrounding structures during a dental trauma, and to evaluate the effect of the mouthguard use to reduce the stress magnitude on these structures. The null hypothesis was that there would be no difference for the stresses generated on the dental surface, regardless of the periodontal ligament health and the MG usage.

## 2. Materials and Methods

**Figure 1:**
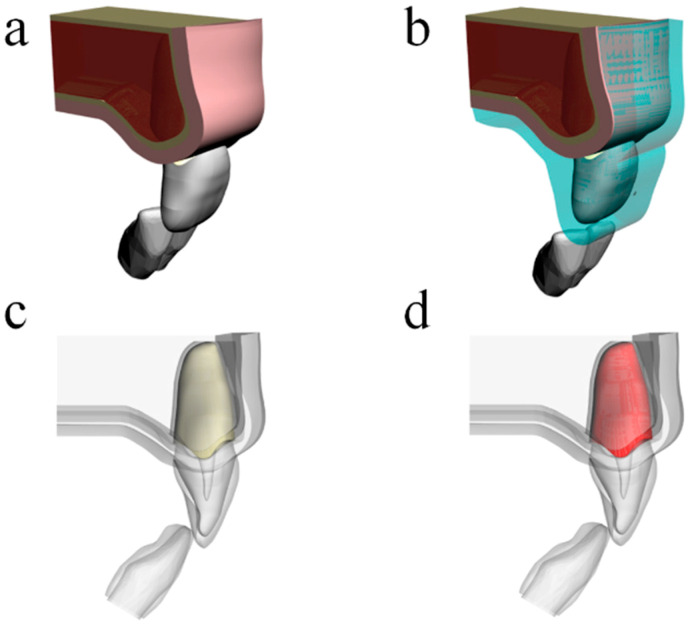
Three-dimensional models simulated in the present study according to: (**a**) absence of mouthguard, (**b**) presence of custom-made mouthguard, (**c**) ankylosed tooth, and (**d**) healthy periodontal ligament.

This study was conducted using a 3-dimensional (3D) finite element analysis (FEA) and a computer-aided engineering software (ANSYS 19.2; ANSYS Inc, Houston, TX, USA) to perform a dynamic structural mechanical analysis. Schematic illustrations of the performed procedures are shown in Figure 1.

For the modeling process, a 3D mathematical model simulating an intact maxillary incisor tooth with supporting tissues was created using computer aided design (CAD) software (Rhinoceros version 4.0 SR8; McNeel North America, Seattle, WA, USA). The model was composed of periodontal ligament and cortical bone with a 0.3- and 1.0-mm thickness, respectively, and medullary bone, enamel, and dentin. In the lateral view, the bone was divided into cortical and medullar sections and then, separated into two juxtaposed geometries, and the hard lamina was modeled following the root anatomy [25]. After that, an custom-made mouthguard model was created based on previous reports [11,13,16] involving 4-mm thickness [15,20], reported as the ideal thickness for this device. For the present study, the mouthguard model was sectioned using cutplanes to be limited to the incisor region following the previous studies’ methodologies. A 35-mm diameter sphere model was created to simulate an impact in the maxillofacial region during the simulation [11,15].

The geometries were exported in Standard for the Exchange of Product Data (STEP) format to a computer aided engineering (CAE) software (ANSYS 19.2, ANSYS Inc. Houston, TX, USA). In sequence, the meshes were generated through the convergence test until obtaining a finite number of nodes and elements for the models. The materials were considered isotropic, homogeneous, and linearly elastic and the mechanical properties used in the simulation are summarized in Table 1 [8,18,24,25,26,27,28,29]. The solids presented perfectly bonded contacts, except the mouthguard that was considered frictionless. For models with dentoalveolar ankylosis, the PDL space was substituted with cortical bone, resulting in direct contact between the root dentin and the cortical bone, as reported by a previous FEA simulation of this condition [5].

**Figure 2:**
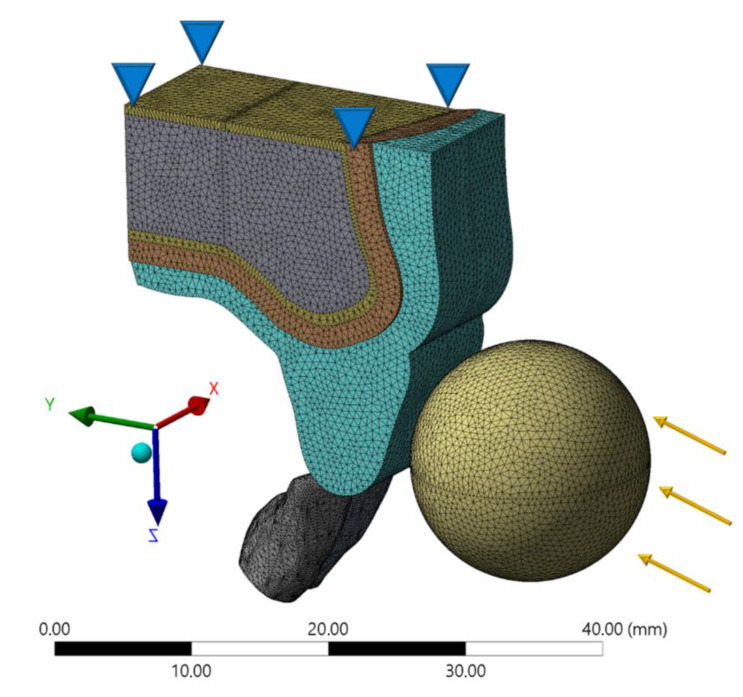
Boundary conditions in the finite element model.

A dynamic impact analysis was performed using a single-step implicit dynamic contact analysis. Boundary conditions defined a rigid impact object (steel sphere) hitting the tooth surface at 1.0 m·s^−1^ initial velocity in the X direction (Figure 2). The impact object was unrestrained in its path after this initial velocity was applied. No gravitational or air-friction forces were modeled, and recoil of the impact object was determined by its inertia and the impact contact responses. The base surface of the maxillary bone was restricted in X, Y, and Z directions [8]. In this study, the stress distribution was analyzed using the von-Mises Stress criteria. Shock absorption capability was defined as the percentage of the stress peak and compared to that of the model without a mouthguard for healthy and ankylosed PDL.

## 3. Results

**Figure 3:**
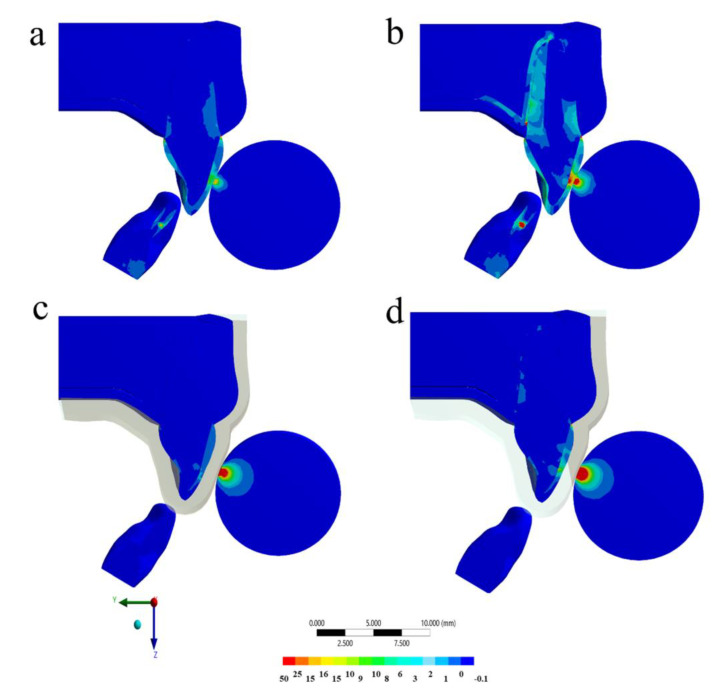
Von-Mises stress distribution in the full model after the impact in different situations: (**a**) Without mouthguard and with healthy periodontal dental ligament (PDL), (**b**) without mouthguard and ankylosed tooth, (**c**) with mouthguard and with healthy PDL, and (**d**) with mouthguard and ankylosed tooth.

**Figure 4:**
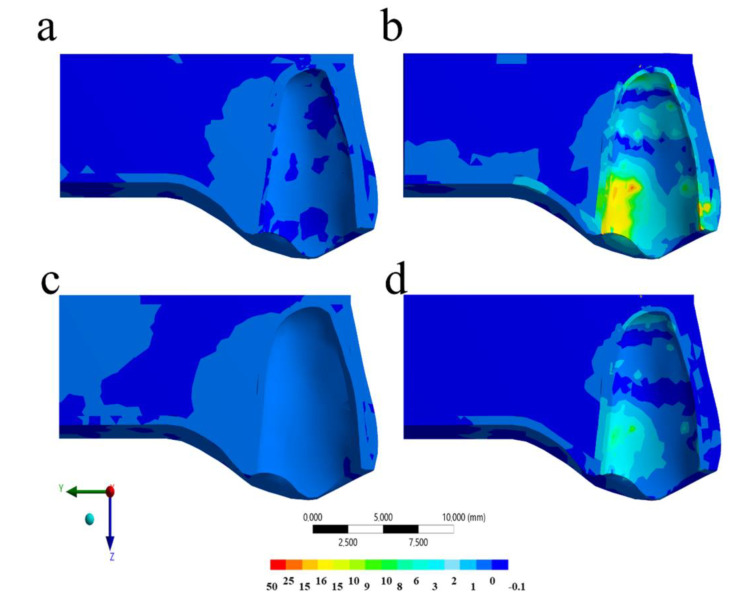
Von-Mises stress distribution in the periodontal dental ligament (PDL) and bone tissue after the impact in different situations: (**a**) Without mouthguard and with healthy PDL, (**b**) without mouthguard and ankylosed tooth, (**c**) with mouthguard and with healthy PDL, and (**d**) with mouthguard and ankylosed tooth.

**Figure 5:**
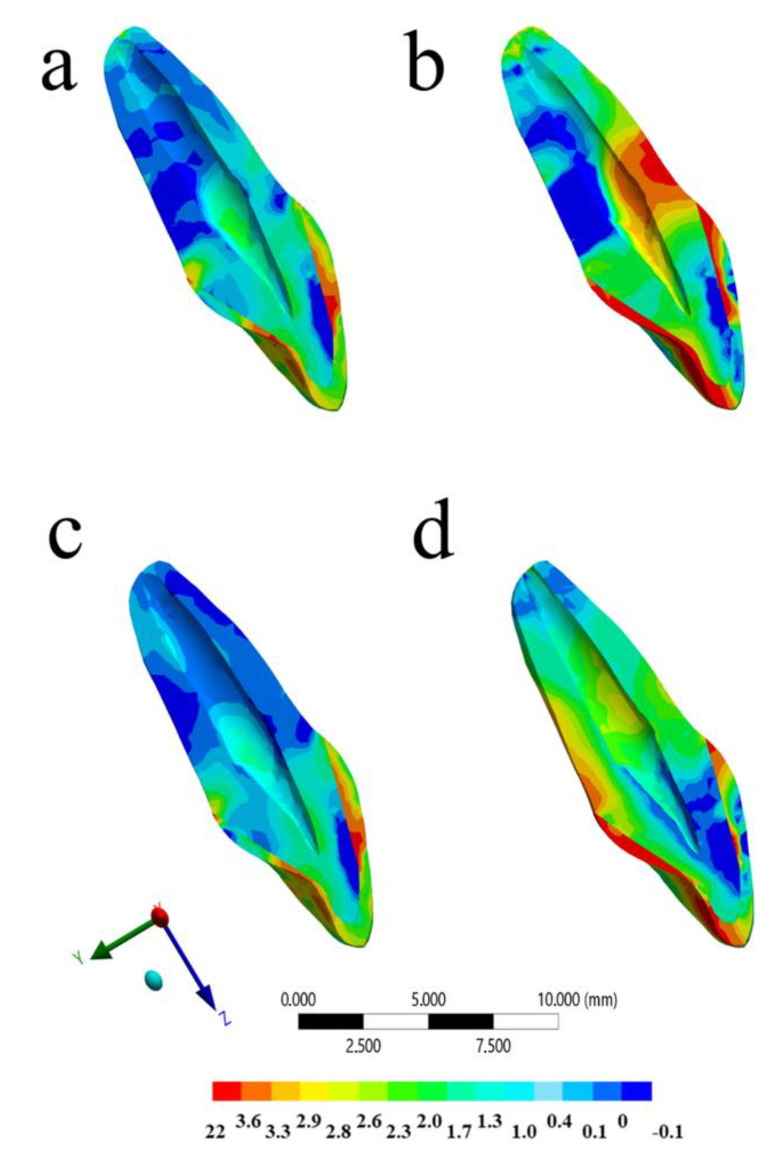
Sagittal view of the Von-Mises stress distribution in the tooth structure after the impact in different situations: (**a**) Without mouthguard and with healthy PDL, (**b**) without mouthguard and ankylosed tooth, (**c**) with mouthguard and with healthy PDL, and (**d**) with mouthguard and ankylosed tooth.

**Figure 6:**
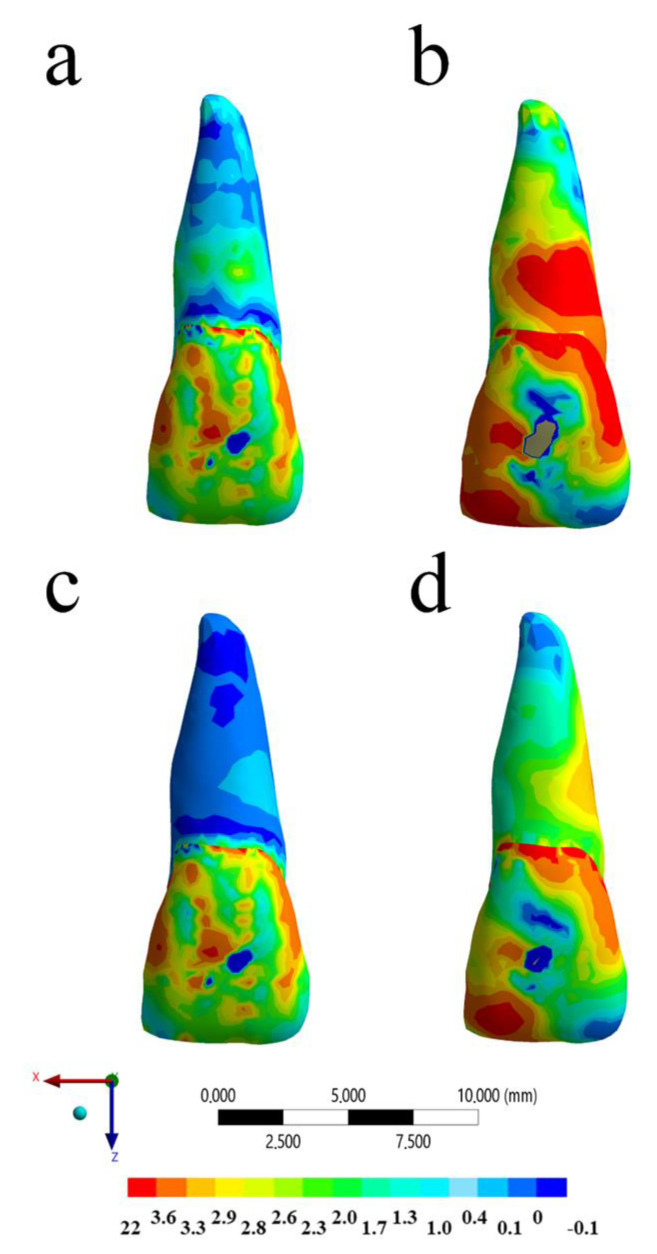
Buccal view of the Von-Mises stress distribution in the tooth structure after the impact in different situations: (**a**) Without mouthguard and with healthy PDL, (**b**) without mouthguard and ankylosed tooth, (**c**) with mouthguard and with healthy PDL, and (**d**) with mouthguard and ankylosed tooth.

The calculated stress can be visualized using a colorimetric scale where blue indicates the lowest stress values and red represents the highest stress values. The results in the maxillofacial structures were determined using the von-Mises stress criteria, correlating tensile and compressive areas. The von-Mises equivalent stress distribution for the different models (with and without MTG, and with and without ankylosed tooth) at the peak of the impact are shown in Figure 3, Figure 4, Figure 5 and Figure 6.

For the PDL tissue, the qualitative stress comparison showed that the highest stress concentrations were at the palatal side and at the cervical buccal region for both models without MG (Figure 3 and Figure 4). However, the higher magnitude was calculated for the ankylosed tooth without MG (Figure 3). It is possible to observe that, for the PDL tissue, the ankylosed tooth with MG in position was still presenting the higher stress concentration than the healthy tooth without this device in position at the impact momentum. Observing the quantitative results for each model, the highest stress magnitude (37.3 MPa) occurred in the situation without mouthguard and ankylosed tooth (Figure 3b), followed by the model with mouthguard and ankylosed tooth (12.5 MPa). For the same impact simulation, the healthy PDL models with and without mouthguard showed the lowest stress at the surrounding tissue (1.8 and 1.9 MPa, respectively).

Regarding the dental structure, the von-Mises maps showed that all the models presented some injury in the buccal face. However, the impact without the MG promoted the worst mechanical response for root dentin and enamel (Figure 5 and Figure 6). In a sagittal view, the model with the ankylosed tooth and without MG showed the highest stress magnitude for the root at buccal face (Figure 5). The presence of MG is able to reduce the stress concentration for the root dentin in a healthy tooth without visible difference for the enamel tissue; however, for an ankylosed tooth, the stress concentration in both tissues can be attenuated with the MG use (Figure 6). Similar to the PDL tissue, the ankylosed tooth with MG still presented a mechanical behavior that is worse than the healthy tissue without MG, suggesting that, even with this device in position, patients with ankylosed tooth are more prone to suffer injury during contact sport. Observing the quantitative results for each model, the highest stress magnitude (22.5 MPa) occurred in the situation without mouthguard and ankylosed tooth (Figure 4b), followed by the model with mouthguard (Figure 4b), and ankylosed tooth (19.5 MPa) in the enamel tissue. For the root dentin, the stress peak was 19.1 MPa without MG and 4.6 MPa with MG for ankylosed PDL. Similar to the previous results, the healthy PDL models with and without mouthguard showed the lowest stress at the root dentin (7.8 and 12.4 MPa, respectively).

## 4. Discussion

The aim of this study was to investigate the influence of dentoalveolar ankylosis on a central incisor and in the surrounding structures during dental trauma, and the effect of mouthguard use in reducing the stress magnitude on the involved structures. The results show that the presence of MG causes a decrease in the stress magnitude in the evaluated structures for both PDL and ankylosed tooth. Therefore, the hypothesis was confirmed.

Different contact sports activities have a high risk of maxillofacial injuries due to falls, collisions with players, hard surfaces, or solid objects [30]. Although often aware of the benefits of using a MG, the sport practitioner do not always use it [31,32,33]. The most reported reasons for not using a MG are the difficulty in breathing, discomfort, talking, or swallowing [31,32]. Similar to previous studies [11,13,15,32], the present report reinforces the need to inform athletes and coaches about craniofacial injuries and the benefits of using a MG during a contact sport activity. The prevalence of orofacial trauma in contact sports practitioners in the Federal District of Brazil is high and most athletes do not use a mouthguard regularly, and some of them do not know about its importance [32].

Although the presence of the MG is important regardless the patient’s occlusion type [32], the present study simulated a condition with the antagonist incisors contacting the bottom surface of the MG. This condition was designed to properly simulate the MG use, since the presence of the antagonist tooth can modify the biomechanical response during the impact. A previous study evaluated the influence of dentoalveolar ankylosis on a single-rooted tooth and the surrounding alveolar bone structures in the biomechanical standpoint using FEA [5]. The authors noted that ankylosed teeth are likely to receive excessive loads during the mastication compared with teeth with normal PDL, justifying the higher stress magnitude in the root dentin and bone. The present study corroborated with this previous study showing a higher stress magnitude for the ankylosed tooth, complementing the previous findings since a dental trauma was simulated and not the chewing forces. It is important to note that the MG use can be beneficial for patients with ankylosed tooth reducing the stress magnitude. However, the generated stress magnitudes for the ankylosed tooth were still higher than the healthy condition, even when unprotected.

According to Jang et al., 2016 [5], a secondary trauma generates bone stress mainly concentrated on the lingual bone crest in tooth with dentoalveolar ankylosis, which might be associated with alveolar bone fracture. However, the authors considered a trauma caused by the antagonist tooth. Based on this, the present study suggests that a trauma caused by an extra-oral object can also increase the risk of tooth and bone fracture.

According to the International Association of Dental Traumatology guidelines for the management of traumatic dental injuries [4], the rate of ankylosis and resorption varies considerably and can be unpredictable. The reasons that lead ankylosed tooth to start a continuous root external resorption is not precisely determined [34]; however, previous authors reported that the injury induces root resorption and may be mechanically induced by dental trauma, surgical procedures, and excessive pressure of an impacted tooth or tumor [2]. Therefore, root resorption can occur without any further stimulus and the bone is laid down instead of the root [2]. The present study suggests that the use of MG in patients with ankylosed tooth should be encouraged during contact activities, especially to avoid mechanical stimuli that could promote further unwanted bone response. Although there are no longitudinal studies showing a higher prevalence of root fracture in ankylosed teeth, the present study suggests that this consequence should be associated with this condition.

There are different types of mouthguards with varying ranges of protection and prices; however, they are all made from polymers and share the same purpose: to absorb and dissipate the impact energy resulting from the shocks [20]. Instead, the mechanical effect of custom-made device is reported as superior and more prone to protect the athletes from injuries due to higher capability to reduce the stress magnitude during an impact [13]. For that reason, the present study simulated a perfectly fitted mouthguard, with uniform thickness and ideal position. However, during the mouthguard manufacturing, in the clinical practice, the mouthguard thickness can be affected by the processing which can affect its appropriate damping effect [12].

The beneficial effect of wearing the mouthguard for sound tooth has been already reported in the literature using three-dimensional [11,13,14] and bi-dimensional analysis [8,16]. All of them showed a reduced stress magnitude for dentin and enamel [8,11,13,14,18]. For bone tissue [8,11,13,20], the MG can also reduce the stress peak at the impact momentum. The periodontal ligament can also present a significant strain reduction when the MG is present [8]. The present study corroborated with all of these previous statements, showing a stress magnitude decrease associated with the MG wearing. For the ankylosed tooth, the MG use reduces the stress magnitude also; however, its biomechanical behavior is still inferior to the sound PDL model.

The literature reports that 31% of orofacial injuries result from sports trauma and 50% are oral and dental injuries. In athletes participating in contact sports, the prevalence of orofacial injuries is 39.1%; however, the type of injury varies based on the sport played, level of competition, the participant’s age, sex, and other factors [35,36]. For example, from professional handball players, 49% experienced head and/or facial trauma and 22% of the participants reported dental injuries [36]. Almost 76% of dental injuries resulted in complications afterward. Sixty-seven percent of the players knew that mouthguards could prevent injuries, but only 28% used them regularly [36].

Some limitations of this study consisted on that the adjacent teeth were not present, the simulated force was applied in the region described in the literature as the most common area related to maxillofacial trauma; however, forces applied in other regions could generate different stress magnitudes [11]. In addition, the mechanical properties were isotropic, which cannot be true for the human tissue. The human tissue was based in an adult maxilla and complete formed central incisor; however, the incidence of dental traumas are very high in children and teenager patients, that can present different bone tissue disposition and mechanical properties as well different and apex formation stages. The mechanical response considering the age relation should be further evaluated too. Another study limitation was the absence of endodontically or prosthetically treatments in the simulated dental element in response to these stresses [37]. Further studies evaluating these factors should be performed in order to elucidate how a prosthetic treated tooth respond to the incidence of dental traumas and how the dental surgeon can reduce the failure risk on it.

However, the results are valid since the limitations are common among the groups and the proportionality observed in the results could possibly be repeated if these conditions were included in further simulations. Different methods can also be applicable to study the mouthguards’ performance, as the pendulum impact [38], since it can serve as complement for further studies regarding the different mouthguard thickness and types.

## 5. Conclusions

The MG use is beneficial for healthy and ankylosed teeth, since it acts by dampening the generated stresses in bone, dentin, enamel and periodontal ligament. However, patients with ankylosed tooth are more prone to root fracture.

## Figures and Tables

**Table 1:** Mechanical properties of the materials used in the computational analysis.

Material/Structure	Elastic Modulus (MPa)	Poisson Ratio	Density (g·cm^−3^)
Enamel [24]	84,100	0.30	2.14
Dentin [26]	18,600	0.30	2.97
Periodontal Ligament [27]	50	0.45	0.95
Cortical Bone [28]	13,700	0.33	2.00
Cancellous Bone [28]	1400	0.31	0.70
Soft Tissue [29]	1.8	0.30	0.95
EVA [8,20]	18,000	0.30	0.95
Steel [8,20]	200,000	0.30	7.80
